# An Evaluation of the Hygiene Practices of Polish Street Food Vendors in Selected Food Trucks and Stands

**DOI:** 10.3390/foods10112640

**Published:** 2021-10-31

**Authors:** Michał Wiatrowski, Ewa Czarniecka-Skubina, Joanna Trafiałek, Elżbieta Rosiak

**Affiliations:** Department of Food Gastronomy and Food Hygiene, Institute of Human Nutrition Sciences, Warsaw University of Life Sciences (WULS), Str. Nowoursynowska 166, 02-787 Warsaw, Poland; michal_wiatrowski@sggw.edu.pl (M.W.); joanna_trafialek@sggw.edu.pl (J.T.); elzbieta_rosiak@sggw.edu.pl (E.R.)

**Keywords:** street food, vendors, hygiene practices, food hygiene, food safety, public health, Poland

## Abstract

Today, street food vending is becoming a dynamically developing food industry in Europe, including Poland. Lifestyle changes and socioeconomic factors, especially among young consumers, make it a convenient food alternative, even in countries without this tradition. The aim of the study was to evaluate hygiene conditions and practices in selected street food facilities in Poland. The study was carried out in accordance with an observation checklist developed on the basis of the hygienic requirements of the European Union. The study covered 550 randomly selected street food vendors in Poland in 10 cities. The hygiene of street food outlets was assessed in three aspects: ensuring proper production conditions, hygiene of production and distribution, and hygiene of personnel. The evaluation of street food outlets showed that the level of hygiene was not fully acceptable. A small percentage of the examined objects satisfactorily fulfilled the requirements of the production conditions, production and distribution hygiene, and staff hygiene. The proposed hygienic checklist for quick daily monitoring of street food outlets can be used to perform internal and external inspections. It seems that specific codes of conduct for European street foods facilities are necessary.

## 1. Introduction

Street food is the sale of food and drink on the street or in other public places, such as parks or shopping malls. It is a food that is ready for immediate or later consumption without the need for further pretreatment or heat treatment. StreetNet International defines it as follows: “A street trader/vendor is someone who sells goods and services on the street pavements or other informal arrangements” [1]. The sale of fresh vegetables and fruit, for example in bazaars or by the roadside, is also considered to be street food [2]. This business can be operated on a fixed or mobile basis, in a market or other public spaces [1]. Street food enables consumers to eat products prepared in a short time and at a low price. The phenomenon of street food is combined with the local culinary culture and eating habits in a given country. It has a positive effect on local economies and the regional ecosystem as it is usually traditional food made from local raw materials. Street food plays an important role in meeting the nutritional needs of urban and small-town residents in many developing and developed countries, as well as tourists. The variety of catering offering street food depends on its location. In developed countries, globalized products such as burgers, fries, hot dogs, and kebabs dominate, but also new, innovative products are introduced, because food trucks have a lower investment risk than restaurants [2,3,4,5,6,7,8]. In turn, in developing countries, food trucks provide niche and traditional dishes, which are easy to prepare from local ingredients [9], as well as international products [10]. Street food is a type of food that is similar to fast food, but the food contains little or no functional additives. On the other hand, street food is more easily accessible to different types of customers, and more natural and traditional, even than slow food restaurants [11]. Street food venues are not as popular in Europe but are also part of many local unique culinary traditions [12,13]. In developed countries, vendors use tailor-made vans (so-called food trucks) and special kiosks that can be transported to another place in one day. In developing countries, stands and exhibition counters dominate [14].

Street vendors are often criticized for delivering low quality goods and creating food safety hazards [4]. According to previous studies on street food, in many countries [7,11,15,16,17,18,19,20,21,22,23,24,25,26,27,28,29,30,31,32,33,34,35,36,37,38,39,40,41,42,43,44,45,46,47,48,49,50,51,52,53,54,55,56,57,58,59,60,61,62,63,64,65,66,67,68,69,70,71,72,73], food production at such outlets is associated with poor or incomplete hygiene of food production and distribution, which possesses a hazard to the health of consumers. This is due to the staff’s lack of hygiene knowledge and lack of training. Finally, more recent studies report ineffective training on hygienic behaviors [30,38]. The microbiological studies indicate the links between street food and foodborne diseases. The studies also indicate that food contamination could be reduced by avoiding common hazards in food production and monitoring of hygiene maintenance rules [21,22,23,24]. 

The aim of this study was to identify and analyze hygiene-related aspects of street food and to estimate food safety risks for Polish consumers. The following research questions were formulated:

− What are the hygiene conditions of food production and distribution in Polish street food facilities?
− Do employees of street food facilities use personal hygiene practices in food production and distribution?

## 2. Literature Review

Street food establishments are most popular in Asia, Africa, and America. These types of facilities are seen as a public health risk due to a weak level of food production and distribution hygiene; studies in Asia [21,22,54,74,75,76,77,78], Africa [23,27,63,79,80,81,82], and America [7,30,41,83,84,85,86,87,88] have shown that they are a public health risk. Street-vended foods have been implicated in outbreaks of foodborne illnesses. Foodborne infections are due to the location of facilities on the street, poor hygienic conditions of food production and distribution, a lack of employee hygiene training, and a lack of required medical examinations of staff. These factors are present both in developed and developing countries around the world [23,41,77,82,89,90]. Among illnesses stated to be the result of foodborne transmission from vendors’ undocumented illnesses [40,91,92,93] are diarrheal diseases [94,95], bacterial contamination, including *Salmonella* spp. [37,74,80], *Listeria monocytogenes* [37], coliforms [75], and hepatitis A virus (HAV) infection. Despite the food safety practices and requirements for street food in developed countries, the number of foodborne illnesses and related risks are rising [96,97,98,99].It is difficult to maintain hygiene in street food establishments due to the large numbers of street food vendors, their diversity, mobility, temporariness, lack of proper infrastructure [100], and the unacceptable condition of street vendors’ facilities [7,76,80]. 

The level of food hygiene in street food outlets varies by country [90,101]. Many studies [23,40,102] have observed a low level of food safety knowledge and poor attitudes to hygiene on the part of street food vendors. Therefore, it is important to use a quick visual assessment of the conditions of food production and distribution by sanitary services. Operators of food trucks and stands should be licensed and monitor the hygiene of personnel, the temperature of meals and proper storage of raw material, and maintenance of sanitary conditions of the production and service area [88]. This is a prerequisite for ensuring appropriate quality and food safety for customers of street food facilities [103]. 

Street food establishments, including those in Poland, have become more and more popular. There are only a few studies on the hygienic condition of Polish and European street food facilities [37,54,104,105]. In the existing literature, there is no comparison of hygienic conditions between food trucks and stands. Our study fills a research gap in the literature on this topic by examining the hygiene of Polish street food venues in selected food trucks and food stands. 

## 3. Materials and Methods

### 3.1. Data Collection

The assessments were performed by a secret inspection and interview. Inspections were the first step of the research. They were carried out by direct observation and the use of an original quick-assessment questionnaire. The inspectors were the authors of this manuscript. We completed HACCP (Hazard Analysis and Critical Control Points) system courses, two of us have an auditor certificate, and our qualifications include topics connected to food hygiene.

Each observation lasted approximately 20 to 30 min and concerned the place of sale and handling practices. The next step was short post-inspection interviews with the employees or owners of street food facilities, which were carried out after the inspection in order to avoid employee awareness of the observation process, so as not to influence their attitudes. The interview was conducted to obtain complementary data. 

### 3.2. Questionnaire Design

A special quick-assessment questionnaire was designed and validated in previous research [51]. The questionnaire was divided into two parts. The first part of the questionnaire was aimed at characterizing the features of each street food outlet, such as the type of premises (stand, food truck), number of consumer seats, number of employees, type of food and beverage offered, and location. The second part of the questionnaire consisted of questions about the hygiene aspects of street food, which were divided into three main sections (Table S1):

- Sanitary conditions of food production (Q.1.1–Q.1.11). This section included 11 questions about the general hygiene condition of the facility where the production processes were performed.
- Hygiene of food production and distribution processes (Q.2.1–Q.2.13). This section included 13 questions related to hygienic production and distribution processes, visual assessment of the quality of raw materials and finished products, as well as the sanitary condition of equipment used.
- Personal hygiene of staff (Q.3.1–Q.3.22). This section included 22 questions related to staff hygiene.

We awarded 1 point for each positive answer to the question about compliance with hygiene requirements. In the first part of the questionnaire, there were 11 questions with a maximum of 11 points, in the second part, there were 13 questions with a maximum of 13 points, and in the third part, there were 22 questions with a maximum of 22 points. 

The following values were assigned to the sum of points in individual sections of the questions and in overall hygiene: 0–50%—unsatisfactory hygiene level; 51–75%—hygiene requires significant improvement; 76–100%—satisfactory hygiene level. The questions were developed on the basis of the requirements of the European Union in Regulation No 852/2004 [106] on the hygiene of foodstuffs and in the *Codex Alimentarius* [107]. 

### 3.3. Characteristics of Street Food Outlets

The study covered 550 randomly selected street food vendors in Poland in 10 large cities: Warsaw (46.3%), Białystok (6.7%), Szczecin (12.9%), Łódź (22.4%), Toruń (1.8%), Kraków (2.7%), Poznań (1.8%), Świnoujście (4.0%), Lublin (0.5%), and Gdańsk (0.9%). The number of inhabitants in these cities ranged from 200,000 to 1,700,000 inhabitants. Each vendor was assigned to one of two groups: stands (30.2%) or food trucks (69.7%). The study was carried out from May to September in 2020 and in 2021. All the outlets were run as microenterprises. Some establishments (65%) operated only in the summer and spring season. The others were open all year round (35%). Some food trucks operated only at large events, the so-called food truck festivals.

Most of the evaluated street food outlets did not have consumer seats (49.5%) or had one or two places for consumers (23%). Three, four, or more consumer seats were found at 27.5% of street food establishments. The number of employees per outlet was as follows: one person (37.9%), two or three people (36.6%), four people (16.4%), or over four people (9.1%). All dishes were prepared in a way that enabled easy and quick consumption and were prepared in front of consumers. Among the types of food offered were: diversified offerings (33.5%); burgers (22.8%); fish (8.2%); kebabs (5.6%); French fries (5.5%); ice cream (4.7%); sandwiches, toast, and casseroles with bread (4.7%); pizzas (3.5%); Tex-Mex cuisine (3.5%); hot dogs (2.6%); desserts, including waffles with various toppings and pancakes (2.0%); sushi (1.4%); vegan meals (1%); and beverages (1%). Meals of five world cuisines were sold in the surveyed outlets: American (29.0%), Polish (12.2%), Turkish (5.5%), European (51.7%), and Asian (1.6%).

### 3.4. Data Analysis

The statistical analysis of the results was performed using Statistica software (version 13.3 PL; StatSoft Inc., StatSoft, Kraków, Poland). 

The results came from the questionnaires, the data were summed and expressed as a percentage of all results provided for each individual section, as well as for overall hygiene. The data obtained in this way were qualitative data. The multiway crosstabulation tables were used for data analysis. The differences between groups were verified with a chi^2^ test (categorical variables). 

However, in the case of the sum of rating (quantitative data), we used the post hoc Fisher test. Before statistical analysis, the normality of the distribution of the variables was checked with a Kolmogorov-Smirnov test. 

Pearson r correlation coefficients were calculated to determine the relationship between the analyzed hygiene factors.

The significance of the differences between the values was determined at the significance level of *p* < 0.05. 

## 4. Results

### 4.1. Sanitary Conditions and Hygiene of Food Production and Distribution in Street Food Outlets

In most elements of the sanitary conditions of food production, the level of compliance with hygiene rules ranged from 50.6 to 76.7% (Table 1). The presence of personal items in the production area was rated as the most common breach of compliance. Only in 50.6% of street food outlets were there no personal items in the production area. Many street food outlets were pest free (76.7% compliance), even though the study was conducted during the summer season. An average level of compliance (65.2%) was recorded in the area of food production and distribution of the surveyed street food facilities. All average scores were at a hygiene level requiring significant improvement in these outlets. 

There were no significance differences found between hygiene of processes of food production and distribution in accordance with the type of street food outlets (food trucks, stands) (*p* > 0.05).

### 4.2. Personal Hygiene of Staff in Street Food Outlets

The results regarding the personal hygiene of staff are presented in Table 2. The personal hygiene of staff was not fully compliant with the hygiene standards (the average level of compliance in different aspects was 59.3%). The lowest average compliance levels (44.8–50.1%) were observed in protective clothing, proper hand washing and drying, and in covering hair while handling food.

### 4.3. Overall Assessment of Hygiene of Street Food Vendors in Poland

The overall hygiene rating of street food vendors in Poland was 63% in compliance with the hygiene requirements (median 29 points of 46 points, Table 3). This result was mainly influenced by the results of the assessments of the personal hygiene of staff (median 13 points out of a possible 22 pts), and hygiene of food production and distribution processes (median 9 points out of a possible 13 points). 

**Figure 1:**
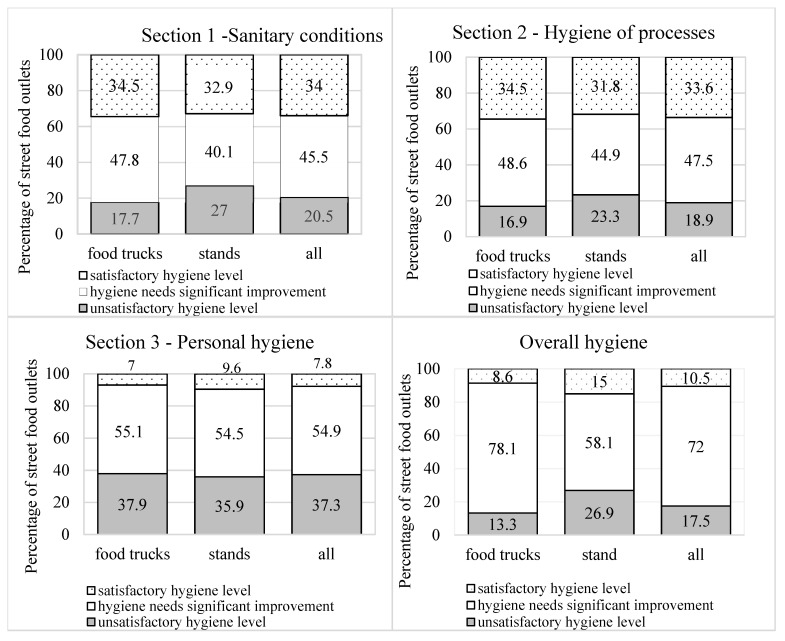
Percentage of street food outlets compliance with different aspects of hygienic rules: 0–50%—unsatisfactory hygiene level, 51–75%—hygiene needs significant improvement, 76–100%—satisfactory hygiene level.

Significantly, more stands (27%) than food trucks (17.8–13.3%) were characterized as having unsatisfactory hygiene in terms of the sanitary conditions of food production (*p* = 0.0415, Figure 1) and in the overall hygiene rating (*p* = 0.00001, Figure 1). 

The conditions of production at the stands and ensuring proper hygiene of production are much more difficult than in food trucks, which are adapted to food production, have appropriate gastronomic equipment, and work surfaces that are easy to maintain hygiene. In a food truck, it is easier to maintain the functionality of the processes including the separation of ‘clean’ and ‘dirty’ processes. In contrast, the stands have a very small working surface, which can lead to cross-contamination. The stands usually do not have constant access to clean water, have little space for storing food, and are also in direct contact with polluted air. Difficult selling conditions may favor the transfer of microorganisms to work surfaces and tools used to preparing and / or serving meals.

In the case of the hygiene of food production and distribution processes, as well as personal hygiene, a similar percentage of food trucks and stands did not comply with hygiene requirements (*p* > 0.05). Only 10.5% (*n* = 58) of all street food outlets had satisfactory overall hygiene, while only 7.8% (*n* = 43) of establishments had a satisfactory level of personal hygiene.

The correlation between inspection results in the observed areas (hygiene conditions of outlets, hygiene of processes, and personal hygiene) and the overall hygiene condition of outlets, has been calculated. A positive correlation between the overall hygiene of street food outlets and hygiene of processes (r = 0.78), personal hygiene (r = 0.78), and sanitary conditions of outlets (r = 0.75), was established. The highest impact on the overall condition of sanitation in a facility was revealed to be the hygiene of food production and distribution processes, as well as staff hygiene.

**Figure 2:**
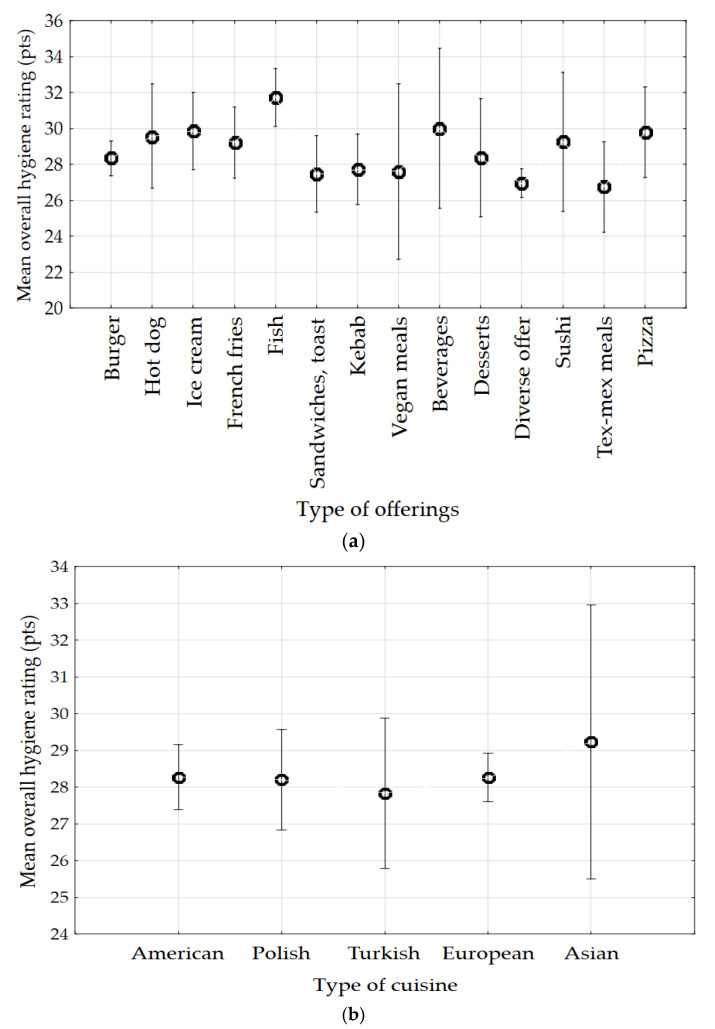
Overall hygiene rating according to: (**a**) type of offerings, (**b**) type of cuisine (bars are standard deviation.

The sum of outlets’ overall hygiene points depended on the type of offer (ANOVA, *p* = 0.0006) (Figure 2). On the other hand, the type of cuisine offered did not affect the overall hygiene rating (ANOVA, *p* = 0.9797). The facilities, regardless of the type of offered cuisines, obtained results at the level of 28–29 points (Figure 2). The best results were obtained by facilities selling fish, i.e., 32 points, which were significantly higher than the results of establishments with burgers (*p* = 0.0004), sandwiches and toast (*p* = 0.0181), kebabs (*p* = 0.0019), diversified offerings (*p* = 0.0324) and Tex-Mex meals (*p* = 0.0010). The lowest results were obtained by facilities with Tex-Mex meals, i.e., 27 points. 

## 5. Discussion

The paper presents the results of research on hygiene requirement fulfillment in European food trucks and stands located in Poland. These facilities offered mainly international meals (e.g., burgers, hot dogs, and French fries), rather than local ones. Polish cuisine was represented by a small percentage (12%) of street food outlets. The examined facilities served city residents, rather than tourists, which is also indicated by other authors [10]. In addition, the types of meal offered by the Polish establishments surveyed differs from those in Africa, Asia, and South America, where it is based on low priced food [7,11,14,21,22,95,108,109,110,111,112,113,114,115,116,117]. Food trucks in Europe are often proprietary mobile facilities for the production and sale of food by famous chefs [12], a local offering addressed to tourists but also products of globalization [9,51]. It is almost impossible to try to characterize the most popular dishes in the gastronomy of both developing and developed countries, due to the diversity in different countries and in the specializations of street food retailers. Some of them specialize in one type of offering, while others have varied offerings [11,47,112,118]. As indicated by reports [119,120,121,122,123,124], in Poland, consumers prefer places serving hot dogs (39.3%), burgers (26.6%), kebabs (22.6%), or ice cream (13.31%), then sandwiches (7.6%), Belgian French fries (7.5%), casseroles with bread (5.8%), or pancakes (4.8%), as well as Asian, Italian, and Tex-Mex cuisine meals, etc., (8.9%). This is in line with the offerings of street food outlets in this study, which underlines the practical importance of the results. 

This paper presented a checklist for quickly assessing the compliance of street food vendors with European Union hygiene standards. The inspection of 550 street food facilities in Poland showed that none of them fully complied with hygiene requirements. Moreover, a significant percentage of street food establishments was characterized by an unsatisfactory hygiene level in all evaluated hygiene aspects. 

Other authors also reported a relatively low percentage (14%) of street food vendors with high levels of compliance regarding sanitation [69]. Similar results of unhygienic food preparation both in food trucks and stands are shown by many other authors [7,19,28,43,45,53,64,125,126,127]. It should be emphasized that a significant number of studies have been carried out in developing countries, where due to the lack of fresh water, limited space for food storage, poor handling of waste generated in technological processes, and a lack of appropriate hygiene of staff, food production takes place with little regard to hygiene for the majority of processes [7,40,41,48,58,63,64,105,115].

On the other hand, the current study was conducted in a European country where HACCP was implemented in food production since 2004. In a significant number of establishments (27%), the sanitary conditions of production were not fully hygienic. This is related to not separating unclean operations from clean ones, overfilled waste containers, dirty floors and walls of preparation areas, contaminated working surfaces, no possibility to clean or disinfect equipment, and most importantly the presence of personal items of the personnel in the production area. Similar non-compliances are mentioned by other authors [78,81,115,128].

Compared to other countries [67], where 51–72% of street food vendors do not apply good hygiene practices, it may be considered an advantageous situation. Trafiałek et al. [54] report similar results. The reason for better hygienic conditions in Poland may be better general education of street food vendors than in developing countries, where only a small percentage of vendors have secondary education and the rest have primary or lower education [7,52,53,65,67,129]. However, some authors suggest that it is not the level of education, but rather training and licensing, that have the effect of increasing food safety knowledge and improving food handling practices [56]. Others [61] showed that vendors’ education is significantly related to their food safety practices. It is not irrelevant that in developing countries, street food is usually an unregulated practice used to solve socioeconomic problems by providing inexpensive food, as well as employment [48]. 

This study found that 40.7% of street food outlets had various non-compliances with personnel hygiene practices. This is consistent with the results of other authors [7,21,22,42,67,77,80,90,95,105,128,130]. As the authors identified, proper and frequent hand washing is particularly important, and, unfortunately, only 40–48% of the vendors they interviewed completed this properly. The reasons for these non-compliances may be related to limited access to sinks and the poor knowledge of staff in this regard. A relatively low level of compliance with hygiene principles was also obtained in terms of the separation of taking payments and technological processes, as well as the use of disposable gloves. Usually, there is only one person employed in a food truck or a stand, who takes care of everything, prepares the food, and takes payments. This type of street food is characterized by haste, as well as connecting to the low hygiene consciousness of personnel, and the lack of hygiene training. Similar non-compliances were indicated by Trafiałek et al. [54,105]. According to the authors, this shows the lack of obligatory training or its insufficient frequency. Non-compliance with hand hygiene is one of the possible causes of secondary contamination of food produced and distributed by street food facilities. 

Sezgin et al. [47] indicate that most countries do not have a street food safety practice. This is true in Poland, where apart from the general requirements for catering establishments, there are no detailed regulations regarding the conduct of this specific type of activity. The Codex Alimentarius is valid all over the world. However, there are no standards or specific patterns for regional products, including street food, and hygiene techniques for their preparation. Future research should focus on the microbiological quality of food production conditions. There are only a few studies in this area. For this reason, it is important to identify which poor hygiene practices are a risk to prepared meals and can cause foodborne illness in people who eat street food. In the interests of public health, it is important to understand the epidemiology of foodborne diseases to help in prevention and in planning control activities. 

### Limitations

Our study had some limitations. First, the study concerned the evaluation of food truck outlets and stands in the European Union. Street food outlets outside this area, e.g., Asia or South America, may have different production and hygiene conditions. Therefore, the questionnaire would need to be adapted to research in other areas. Secondly, the study concerned facilities located in cities, where the availability of infrastructure such as water and electricity necessary for cold storage and washing processes may be better than in the countryside. Another limitation was the lack of microbiological tests that would give a complete picture of the hygiene condition of food trucks and stands, as well as the employees. Finally, the proposed special fast evaluation questionnaire would need to be modified in the case of the evaluation of outlets from other industry sectors.

## 6. Conclusions

The conducted research showed that the level of hygiene in the assessed Polish street food outlets was not fully satisfactory. Only a small percentage of the examined businesses satisfactorily met hygiene requirements in terms of production conditions, production and distribution hygiene, and staff hygiene. It should be emphasized that the worst results were obtained in the area of staff hygiene, which was mainly dependent on training. Given the growing interest in street food outlets among European consumers, for the sake of public health, it is necessary to carry out a quantitative or semi-quantitative risk analysis of this type of food offering.

Deficiencies in compliance with the basic rules of food safety indicate that despite the applicable regulations and mandatory hygiene requirements, new solutions should be sought. A solution to these problems may be to use a daily hygiene quick checklist made by staff at the start of the working day and unannounced inspections by owners. This will allow for an effective quality risk assessment of hygiene practices and conditions and potentially provide consumers with safe street food. A checklist can be used for the inspection of street food outlets by external auditors, as well as a diagnostic tool for internal audits by street food owners. The results of the study can help in planning training programs for people starting this type of business in order to develop appropriate hygiene attitudes and practices. They can also be used to develop specific mandatory codes of conduct for the production and distribution of street food establishments in Europe. This study contributes to the body of literature on hygiene and food safety practices of street food vendors operating in Poland and other European countries. 

## Figures and Tables

**Table 1:** Sanitary conditions and hygiene of food production and distribution in street food outlets (*n* = 550).

Section	Question	Areas of Food Production *	Compliance	Noncompliance
			with Hygienic Rules (% of Outlets)	
Section 1	1.1	Hygiene of production area	73.2	26.8
	1.2	Functionality of production process	70.3	29.7
	1.3	Condition and cleanliness of waste bin	71.9	28.1
	1.4	Condition and cleanliness of floor	66.5	33.5
	1.5	Condition and cleanliness of production tops	71.2	28.8
	1.6	Condition and cleanliness of facility walls	63.6	36.4
	1.7	System of air ventilation	62.1	37.9
	1.8	Food pests in the production area	76.7	23.3
	1.9	GHP ** at the facility	60.7	39.3
	1.10	Possibility to clean/disinfect the equipment	65.9	34.1
	1.11	Personal items in the production area	50.6	49.4
		**Average level of compliance in Section 1**	**66.6**	**33.4**
Section 2	2.1	Condition of working places	67.6	32.4
	2.2	Separation of food from the consumer	71.4	28.6
	2.3	Correctly using and storing the packaging and tableware	64.1	35.9
	2.4	Proper raw material storage	73.6	26.4
	2.5	Separation of raw material and final products	54.1	45.9
	2.6	Separation of ready-to-eat products and waste	66.3	33.7
	2.7	Use of separate equipment during meal preparation and distribution	61.2	38.8
	2.8	Condition and cleanliness of catering equipment	69.8	30.2
	2.9	Proper handling of tableware	62.8	37.2
	2.10	Unauthorized people in the production areas	60.1	39.9
	2.11	Quality of semi-finished and ready-to-eat products	72.7	27.3
	2.12	Possibility for cross contamination activities	60.5	39.5
	2.13	Hygienically handling packaging and tableware	63.6	36.4
		**Average level of compliance in Section 2**	**65.2**	**34.8**

* References to criteria of assessment regarding Regulation (EC) 852/2004. ** GHP—Good Hygienic Practice.

**Table 2:** Personal hygiene of staff in street food outlets (*n* = 550).

Question	Aspects of Personal Hygiene of Staff *	Compliances	Non Compliances
		with Hygienic Rules (% of Outlets)	
**3**	**Section 3**		
3.1	Cleanliness of employee’s hands	72.9	27.1
3.2	Cleanliness of employee’s nails	64.5	35.5
3.3	Hand jewelry wearing by workers	59.2	40.8
3.4	Correct protection from hand injuries	68.1	31.9
3.5	Compliance with no-drinking and no-eating rules in the production area	55.9	44.1
3.6	Correct working attire of employees	62.5	37.5
3.7	Workers’ personal attire covering	44.8	55.2
3.8	Correct hand-washing by workers	50.1	49.9
3.9	Correct hand-drying after washing by workers	46.6	53.4
3.10	Sink for washing hands with instructions in the production area	57.9	42.1
3.11	Proper accessories to wash hands in facilities	57.7	42.3
3.12	Separation of handling money from handling food	57.6	42.4
3.13	Proper use of disposable gloves by workers	57.7	42.3
3.14	Cleanliness of staff hair	69.0	31.0
3.15	Correct hair covering of workers during food handling	50.1	49.9
3.16	Long hair protection by workers	55.4	44.6
3.17	Private clothing items used by workers	56.3	43.7
3.18	Presence of earrings or other facial accessories on staff during work	58.1	41.9
3.19	Head or skin problems of workers	71.2	28.8
3.20	Illness of workers	66.7	33.3
3.21	Excessive make-up on workers	61.0	39.0
3.22	Touching face, hair, nose, or ears during food production by workers	60.7	39.3
	**Average level of compliance in Section 3**	**59.3**	**40.7**

* References to criteria of assessment regarding Regulation (EC) 852/2004 [106].

**Table 3:** Results of the overall evaluation of street food vendor hygiene.

Assessed Area	Type of Outlet	Maximum Points	Grade (Points)		*p*-Value *
			Average ± SD	Median	
Sanitary conditions of food production	All outlets Food trucks Stands	11	7.3 ± 2.2 7.5 ± 2.1 7.0 ± 2.3	8 8 7	0.0419 *
Hygiene of processes of food production and distribution	All outlets Food trucks Stands	13	8.5 ± 2.2 8.6 ± 2.1 8.3 ± 2.4	9 9 9	0.1225 *
Personal hygiene of staff	All outlets Food trucks Stands	22	12.4 ± 2.9 12.5 ± 2.8 12.3 ± 3.3	13 12 13	0.5779 *
Overall hygiene rating	All outlets Food trucks Stands	46	28.2 ± 5.7 28.5 ± 5.1 27.6 ± 6.9	29 29 29	0.0049 *

SD—standard deviation, *—between type of street food outlets (food trucks and stands).

## Data Availability

The data presented in this article are available on reasonable request from the corresponding author.

## Supplementary Materials

The following are available online at https://www.mdpi.com/article/10.3390/foods10112640/s1, Table S1: Questionnaire structure.
