# Supplementary material for: An Evaluation of the Hygiene Practices of Polish Street Food Vendors in Selected Food Trucks and Stands
Source: Foods. 2021 Oct 31;10(11):2640. doi: 10.3390/foods10112640 (PMC8622227; doi:10.3390/foods10112640)
Supplement: Supplementary file 1 [file foods-10-02640-s001.zip › foods-1426453-supplementary.pdf]

foods

MDPI

Table S1. Questionnaire structure

Number	Questions *	Compliances with Hygienic Rules (Yes or No)
1	**Section 1: Sanitary conditions of food production**	
Q.1.1.	Is the production area hygienic?	
Q.1.2.	Are the production processes functional (dirty operations are separated from clean) at the facility?	
Q.1.3.	Is there a waste bin in the production area and is it overflowing?	
Q.1.4.	Is the floor in a good condition (clean, undamaged, easy to wash and disinfect)?	
Q.1.5	Are the production tops clean, smooth and made of a material that is easy to clean and disinfect?	
Q.1.6	Are the facility walls clean, washable, non-absorbent and non-toxic?	
Q.1.7	Is there a system of air ventilation at the facility?	
Q.1.8	Are there any food pests in the production area? (visually determined)	
Q.1.9	Is the GHP implementation possible at the facility?	
Q.1.10	Is it possible to clean/disinfect the equipment?	
Q.1.11	Are there no personal items (phone, bag) in the production area?	
2	**Section 2: Hygiene of processes of food production and distribution**	
Q.2.1	Are the working places in a good hygienic condition?	
Q.2.2	Is the food separated from the consumer in a way that excludes a secondary contamination?	
Q.2.3	Are the packages, tableware clean, in a good condition and in a storage disabling their contamination?	
Q.2.4	Are the raw materials stored in a proper temperature?	
Q.2.5	Is there a separation of raw material and final products?	
Q.2.6	Are the ready-to-eat products and waste in separation?	
Q.2.7	Is there a separation of catering equipment during the preparation and distribution of meals?	
Q.2.8	Is the catering equipment clean and in a good condition (visually determined)?	
Q.2.9	Is there a space for returning tableware or a waste bin for the disposable tableware?	
Q.2.10	Are there no unauthorized people in the production areas?	
Q.2.11	Is the quality of semi-finished and ready-to-eat products satisfying (freshness visually determined)?	
Q.2.12	Are there no production activities where the clean and dirty areas may cross one another?	
Q.2.13	Do workers handle packaging, disposable plates and cutlery hygienically?	
3	**Section 3: Personal hygiene of staff**	
Q.3.1	Does the employee have clean hands during work?	
Q.3.2	Does the worker have clean, short, unpainted and natural (no tips) nails?	
Q.3.3	Does the worker not wear hand jewelry during work?	
Q.3.4	Is there a correct treatment and protection of the injuries of hands?	
Q.3.5	Do workers comply with no-drinking and no-eating rules in the production/ distribution area?	
Q.3.6	Do workers have correct working clothes?	
Q.3.7	Is the personal clothing protection covering all of the worker's personal clothing?	
Q.3.8	Is the worker washing his hands properly and frequently (during the observation)?	
Q.3.9	Is the worker drying hands after washing correctly (during the observation of his work)?	
Q.3.10	Is there a sink for washing hands with instruction of hand washing & disinfection in the production area?	

Q.3.11	Are there all of the proper accessories near the sink (i.e. soap, paper towels, waste bin)?
Q.3.12	Is taking fees properly separated from the production ?
Q.3.13	Do workers have and change disposable gloves frequently enough?
Q.3.14	Does the staff have a clean hair?
Q.3.15	Is the staff covering their hair during food work?
Q.3.16	Are workers protecting their long hair (thus minimalizing the risk of food contamination)?
Q.3.17	Do workers have personal clothing items in addition to working clothes?
Q.3.18	Does the worker not have earrings or other accessories on his head during food work?
Q.3.19	Does the staff not have skin problems of the head?
Q.3.20	Does the staff not have an illness (cough, sneeze) that makes hygienic work difficult?
Q.3.21	Does the staff not have excessive make-up?
Q.3.22	Are the workers not touching their face, hair, nose, or ears during food production?

* References to criteria of assessment regarding Regulation (EC) 852/2004.
